# Aging of the inceptive cellular population: the relationship between stem cells and aging

**DOI:** 10.18632/aging.100036

**Published:** 2009-04-02

**Authors:** Catherine E. Symonds, Umberto Galderisi, Antonio Giordano

**Affiliations:** ^1^ Sbarro Institute for Cancer Research and Molecular Medicine, Center of Biotechnology, Temple University, Philadelphia, PA 19122, USA; ^2^ Department of Experimental Medicine, Section of Biotechnology and Molecular Biology, Excellence Research Center for Cardiovascular Diseases, Second University of Naples, Naples, Italy; ^3^ Department of Human Pathology and Oncology, University of Siena, Siena, 53100, Italy

**Keywords:** stem cells; senescence; cell cycle; apoptosis; aging; differentiation

## Abstract

The average life expectancy worldwide has about doubled and the global population has increased six fold over the past century.  With improving health care in the developed world there is a proportional augmentation in the treatment necessary for elderly patients occasioning the call for increased research in the area of aging and age-related diseases.  The manifestation of this research has been focalized on the causative cellular processes and molecular mechanisms involved.  Here we will discuss the efforts of this research in the area of stem cells, delving into the regulatory mechanisms and how their de-regulation could be attributed to aging and age-related diseases.

## Introduction

The complexity of mammalian development is intrinsic to the zygote with genes encoding the information necessary for every tissue and cellular sub-type. Development initiates with totipotent embryonic stem cells (ESCs), which give rise to the three germ layers, the ectoderm, the mesoderm and the endoderm, eventually constructing the tissues of the body.  ESCs possess characteristics such as asymmetric cellular division,  the ability to  differentiate into all three germ layers, telomerase activity, and a cell cycle that has significantly diminished gap phases.  In adulthood, multipotent tissue specific stem cells regulate homeostatic tissue regeneration.  These adult stem cells (ASCs) while lacking the capacity to differentiate into the three germ layers are capable of regenerating a cellular popula-tion of a specific tissue type and maintain asymmetric cellular division.  ASCs are characterized as being in a state of relative proliferative quiescence, which they can exit from under the proper conditions, to obtain the proliferative potential necessary for tissue regeneration.

ASCs are primarily responsible for maintaining tissue structure; they accomplish this through their functional duality between self-renewal and commitment.  The tissue specific ASC populations are vital to survival and therefore must be maintained through self-renewal.  However, the necessity for self-renewal must be transient, as the cells are also required to differentiate and commit to a specific lineage.  The balance between self-renewal and commitment is critical.  If the ASC population leans towards the self-renewal pathway, it risks the loss of differentiation capacity and could malignantly transform into a highly proliferative nondifferentiating cellular population.  On the other hand, if the balance shifts towards differentiation, there is a risk that the stem cell population would be lost accompanied by an increased potential for degenerative disease occurrence, a mechanism that is believed to be a component of the aging process.

Organismal aging and age-related diseases are often associated with senescence. Hayflick originally described senescence as a permanent cell cycle arrest due to the limited replicative potential of cultured human fibroblasts [1].  Telomere shortening, oncogene activation or DNA damaging events can trigger the senescence pathway.  Senescence plays a critical role in maintaining properly functioning ASC populations.  Under normal conditions ASCs divide to replace aging tissue. During lifetime, extrinsic sources (such as ionizing radiation, genotoxic drugs, chemicals, etc.) and intrinsic factors (DNA replication errors, spontaneous chemical changes to DNA, programmed DNA recombination) can lead to mutations, which could accumulate over time in the progenitor population.

Regeneration can also be triggered due to tissue damaging events, which could directly expose the ASC population to mutations and/or alter the regulatory tissue microenvironment.  Stem cells possess inherent damage repair mechanisms that can respond to DNA-damage, reactive oxygen species (ROS) damage and mutations that de-regulate the cell-cycle and other cellular functions.  When these repair mechanisms fail the cell will accrue increased levels of damage, initiating cell-death pathways such as senescence or apoptosis (programmed cell death). The depletion of the progenitor cell population results in an inability for tissue renewal, aging and possible development of degenerative diseases.  On the other side, failing to properly repair DNA damage along with escape from apoptosis and/or senescence could trigger neoplastic transformation of stem cells.

Stem cells encapsulate such an immense clinical therapeutic potential that understanding their intricate biological role is paramount.  Unfortunately, the definition of stem cells remains to be nebulous and data can be contradictory.  In this review we will attempt to describe stem cell properties in both embryonic and adult stem cells and the intriguing regulation of the cell cycle in these systems.  We then discuss the role of the senescence process in ASCs and its relation to aging and age-related diseases. Concluding, we will examine how the de-regulation of the mechanisms discussed may lead to carcinogenesis and what stem cell research may hold for future therapeutic prospects.

### Embryonic stem cells and their origins

Thomson and Gearhart are attributed with initial isolation and characterization of human ESCs (hESCs) from the inner cell mass of the blastocyst, noting the differentiation and self-renewing capacity of the cells *in vitro* [2,3]. Further characterization demonstrated that the cells expressed cell surface markers typical of undifferentiated nonhuman primate ESCs (pESCs) and human embryonic carcinoma cells as was originally described [2,3,4,5].  These specific markers included stage-specific embryonic antigen (SSEA)-1, SSEA-3, SSEA-4, TRA-1-60, TRA-1-81, alkaline phosphatase activity and high levels of telomerase activity [2,3].  Telomerase is a ribonucleoprotein enzyme that preserves the telomeric regions at the ends of chromosomes by *de novo* oligonucleotide synthesis [6].  Telomerase activity is not present in normal diploid somatic cells, which incur shorted telomeres with age leading to replicative senescence after a finite number of replications [1,7,8,9].  It has been shown that TRA-1-60 and TRA-1-81 are specific epitopes of a larger membrane-bound protein podocalyxin, which under-goes retinoic acid modification when ESCs differentiate losing its reactivity with the TRA-1-60 and TRA-1-81 antibodies [10].  These characterizations remain to be used to identify stem cells today, along with the expression of the intrinsic transcription factor Oct-4 and in mouse ESCs (mESCs) the constitutive ability to receive extrinsic signals from the cytokine leukemia inhibitory factor (LIF) [11,12,13].

Soon after the initial isolations and characterizations of hESCs, interest shifted towards understanding the factors involved in their differentiation. For example, if all cells are derived from initial progenitor cells, what directs differentiation towards glial cells versus adipocytes?  Brüstle et al were among the first to demonstrate *in vitro* controlled differentiation of hESCs using a series of growth factor combinations, which successfully elicited a reactivity to a monoclonal antibody specific for a membrane epitope typically found on the membranes of glial precursors [14].  They initially grew ES cells in a media that favored the growth of neural precursors.  They then exposed cells to the following series of growth factors: i) basic fibroblast growth factor (FGF2), ii) FGF2 and epidermal growth factor (EGF) and iii) FGF2 and platelet-derived growth factor (PDGF) [14].  The cells maintained in the final growth factor-supplemented media were able to be stored and kept in culture without further differentiation for many passages.  However, as growth factors were removed cells further differentiated into more specific neural cell types such as oligodendrocytes and astrocytes [14].  The cells that were preferentially differentiated were injected into a rat model of a human hereditary myelin disorder, Pelizaeus-Marzbacher disease, and effectively remyelinated the axons of the brain and spinal chord [14].  These results as well as others [15,16] demonstrated the potential to manipulate the differentiation of isolated hESCs *in vitro* for therapeutic treatment of human disease.

### Embryonic stem cells and their regulation of the cell cycle

A major difference between stem cells and somatic cells is found in the basic regulation of the cell cycle.  In somatic cells the cell cycle is controlled mainly by Rb-E2F family complexes, cyclin-cyclin dependent kinases (Cdks), and Cdk inhibitors through the INK4a/ARF pathway.  Undulations in expression and post-translational modifications of the proteins involved in these pathways result in the control and regulation of the cell cycle.  Likewise, mutations or de-regulation of these proteins can lead to uncontrolled cell proliferation, aneuploidy, and genomic instability [17,18].

The cell cycle regulatory mechanisms, which differ between somatic cells and ESCs have been determined using the mESC model in combination with mESCs representing a pluripotent lineage (mEPLC) [19].  mESCs of late pre-implantation and early post-implantation embryos proliferate at an unusually rapid rate [20].  Between 4.5 and 6.0 dpc (days post coitum), the epiblast expands with a generation time of approximately 10 hours [21].  This increases between 6.5 and 7.0 dpc, where mean generation times are found to be approximately 4.4 hours [21,22].  The cell cycle in mESCs and mEPLCs has been found to curtail G1 and G2 phases with an increased proportion of the cycle, approximately 50-60%, spent in S phase [23,24].

**Figure 1.**
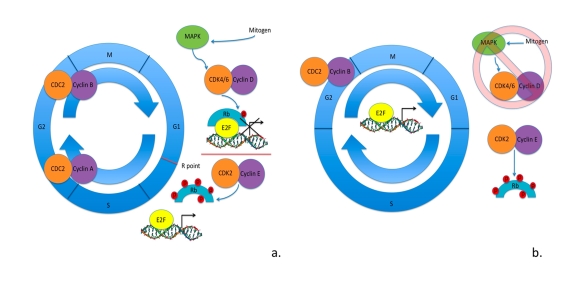
Cell cycle in somatic cells vs. ESCs. (**a**) Cell cycle regulation in somatic cells: mitogen signaling through MAPK pathway activates cyclin D - CDK4/6 kinase activity hypophosphorylating Rb family member proteins. Hypophosphorylated Rb family member proteins bind to E2F transcription factors blocking the transcription of E2F-regulated genes.  To surpass the R point cyclin E - CDK2 kinase activity is activated hyperphosphorylating Rb family member proteins.  Hyperphosphorylated Rb family member proteins are unable to interact with E2F factors, allowing them to activate transcription of genes necessary in the progression of cell cycle. (**b**) Cell cycle regulation in ESCs as is currently understood.  Mitogen signaling through MAPK pathways seems to be irrelevant in the progression of cell cycle.  There is cell cycle-independent expression of cyclin E - CDK2 maintaining the hyperphosphorylated levels of Rb family member proteins.  This results in cell cycle-independent expression of E2F-regulated genes.  Cyclin B - CDC2 is the only CDK activity that appears to be regulated by the cell cycle.  ESCs have shortened gap phases and an elongated S phase of the cell cycle, with an apparent lack in the R point for G1-S transition.

Under normal somatic cell cycle conditons, Rb/p105, in the hypophosphorylated state, interacts with E2F transcription factors inhibiting the transcription of genes necessary for the progression of the cell cycle through the restriction point (R point).  The phosphorylation levels of Rb/p105 are dependent upon the CDK activity present in the cell.  Mitogen signaling through the Ras/Raf/mitogen activated protein kinase (MAPK) pathway activates the cyclin D - CDK4/6 complexes, which are believed to initially activate Rb/p105 activity by hypophosphorylating the unphosphorylated protein.  To pass the R point of the cell cycle cyclin E/CDK2 hyperphosphorylates Rb/p105 inhibiting the protein from binding to E2F transcription factors thus initiating the transcription of genes required in the S phase of the cell cycle.  To obtain a cell cycle that is less influenced by mitogen variations, stem cells appear to adopt a different regulation mechanism as depicted in Figure 1 [25,26].

Along with shortened gap phases in the ESC cell cycle, the R point does not seem to regulate the G1 - S transition. Stead and collegues found that in both mESCs and mEPLCs there was a precocious cell cycle-independent expression of CDK2, cyclin A and cyclin E kinase activity [24].  Furthermore, when CDK2 was suppressed they found a significant decrease in cell proliferation rate. Instead CDC2 - cyclin B, essential to G2 - M transition, was the only CDK activity that was found to be cell cycle-dependent and E2F target genes were constitutively expressed throughout the cell cycle [24].  Evidence has also shown a lack in hypophosphorylated Rb/p105, instead findings support the presence of hyperphosphorylated Rb/p105 in mESCs and mEPLCs [23,27].  Given the cell-cycle independent expression of cyclin E and CDK2, it would be logical that Rb/p105 would be found in the hyperphosphorylated state, further supporting the absence of the R point in ESC cell cycle progression (Figure 1).

Mitogen signaling through the MAPK pathway normally stimulates cell division in somatic cells, however, this signaling when prolonged is a potent inducer of differentiation.  mESCs appear to avoid this stimulation by maintaining low levels of cyclin D expression and almost no detectable CDK4 kinase activity [28].  This corresponds to the lack in hypophosphorylated Rb/p105 levels previously detected in mESCs.  These findings support the absence of early G1 in mESCs, allowing them to avoid the differentiation-inducing effects of MAPK signaling as is found in other cell types.

Although the majority of studies thus far have been performed in mESCs, hESCs similarly show a truncation of the G1 phase of the cell cycle, however not much else is known about cell cycle regulation in hESCs.  Interestingly, primate ESCs behave similarly to mESCs in having cell cycle-independent expression of cyclin E, constitutive hyperphosphorylation of Rb/p105 and serum and MAPK-independent cell cycle progression [28,29].  Therefore, it could be inferred that through conserved evolution hESCs may regulate the cell cycle in a similar fashion. Taken together these data lead to the hypothesis that the ESC cell cycle is rate-dependent upon high levels of CDK activity, is not regulated by Rb/p105 or E2F gene expression, lacks the G1 check point and the traditional periodicity found during the somatic cell cycle.

### Adult stem cell characteristics

The first evidences of adult stem cells were described as lympho-haematopoietic stem cells, which were capable of giving rise to both erythroid and lymphoid  progeny [30].  The previous medical studies, showing the capability of bone marrow to regenerate a transplanted patient's bone marrow attributed credibility to these finding [31].  Presently, adult stem cell tissue regeneration is not a foreign concept and it is well accepted as the regenerative mechanism in tissues such as the intestinal epithelium, bone marrow, and skin.  The almost constant regeneration of these tissues has been linked to tissue specific adult stem cell populations, which when deregulated have been associated with various diseases and cancers [32,33,34,35].  While these were the most physiologically obvious tissues in which stem cell regeneration could occur, adult stem cell populations have also been identified and characterized in the retina [36], the pancreas [37], the liver [38], the central nervous system [39], and in skeletal muscle [40].

The most heavily studied populations of ASCs are the haematopoietic and mesenchymal stem cells (HSCs and MSCs).  HSCs are the progenitor lineage that produces all of the mature blood cells throughout an organism's life.  It was originally noted that the HSC population contained two populations of stem cells, which responded differently to radiation [41].  The cycling population was unable to repair DNA-damage and produced acute marrow failure, whereas the more primitive quiescent population appeared to repair radiation induced damage [41].  These findings have been further supported and outline the classification of HSCs into the two following groups: the long-term repopulating HSCs (LTR-HSCs), primarily found in a quiescent state, and the short-term repopulating HSCs (STR-HSCs), which undergo haematopoiesis supplying the daily replenishment of mature blood cells. This mechanism that HSCs have adopted has allowed the progenitor population of LTR-HSCs to maintain genomic integrity by reduced replication events [42].  MSCs are a cellular population found in the bone marrow alongside the HSCs, which differentiate into cells of the mesenchymal lineage including bone, cartilage, fat, connective tissue, muscle and marrow stroma [43,44].  The MSC population is quite heterogeneous and more recently multiple pre-MSC lineages have been described: MAPC, hBMSC, USSC, FSSC, AFS, MIAMI cells, hFLMPC, and MASC [45,46,47,48,49,50,51,52].  Pre-MSCs have been shown to differentiate and form the three germinal layers, furthermore multiple lineages have been shown to proliferate without telomere shortening [42].  These lineages speak to the complexity of the regenerative mechanism that still has yet to be well defined.

Aside from haematopoietic and mesenchymal stem cells (HSCs and MSCs) the identified adult stem cell populations have been onerous to study, due to the difficulty in isolating and culturing the cells *in vitro* [35].  Since their discovery, it has been understood that adult stem cells reside in niches that supply the cells with necessary growth factors and stimulation to undergo self-renewal and proliferation [53,54].  When these growth factors are applied to *in vitro* culture conditions, viable adult stem cell culture has been achieved [55,56,57,58,59,60].  However, there is still restricted understanding of these adult stem cell populations and their properties.

Genomic studies, utilizing microarray technology, have identified molecular signatures for specific and across diverse populations of stem cells [61,62,63].  Particular genes were found to span both ESCs as well as diverse adult stem cell lineages.  These studies concluded that while many of these genes were ubiquitously expressed in other tissues, a subset of this grouping could represent genes involved in general stem cell growth and maintenance [35].  More recently, Rossi et al. using an oligonucleotide microarray, identified 907 out of 34,000 genes that were significantly differentially expressed between HSCs from young and old mice, sixteen of the genes more highly expressed in older animals have been implicated in human leukemia.  [64,65,66].  A similar study found that genes of the functional categories DNA repair, chromatin remodeling, and silencing genes were expressed less in HSCs from aged animals.  These findings may suggest genetic and epigenetic alterations that are responsible for the differences observed between young and old HSCs.

### Adult stem cells and their regulation of the cell cycle

Adult stem cells, differing from ES cells, maintain a quiescent state *in vivo* unless they are stimulated by tissue damage or regenerative signals to differentiate.  In normally dividing cells entering into the quiescent state there is an upregulation of CDK inhibitors, which act to block the kinase activity of CDKs effectively blocking cell proliferation [67,68,69,70,71]. Further-more, CDK inhibitor expression is independently sufficient to inhibit proliferation [67].  CDK inhibitor expression is found in quiescent ASCs and when downregulated can initiate proliferation and differentiation in HSCs [72,73,74].

Unfortunately, due to the limitations in the data that are currently available, it is not possible to construct a detailed ASC cell cycle model.  From what has been gleaned of the ESC model, it is believed that the G1 phase and the R point are critical in the decision between self-renewal and differentiation, as well as the directionality of differentiation.  Much of the data seems to suggest that the mechanisms regulating the cell cycle are extrinsically supplied from the cellular microenvironment, the niche.  However, more studies will be needed before we can truly understand the roles of the regulatory protein mechanisms recognized for so long as the classical cell cycle model.

### Aging and maintenance of adult stem cells

Self-renewal of stem cells is critical for their persistence through life, however the capacity to maintain this characteristic declines with age [75,76].  The decline in the maintenance of the self-renewal pathway is considered one of the major mechanisms attributing to aging.  p16^Ink4a^, a cyclin-dependent kinase inhibitor, promotes Rb/p105 activation and is associated with the triggering of the senescence pathway [77].  It has more recently been ascribed to stem cell aging and loss in self-renewing properties [75,78].  In fetal stem cell populations there is no detectable expression of p16^Ink4a^, however increasing levels of p16^Ink4a^ expression have been detected in stem cells of aging tissues [76,79].

The*INK4a/ARF* tumor suppressor locus encodes for p16^Ink4a^ and p19^Arf^, which act respectively through the Rb and p53 cell death pathways [80].  The *INK4a/ARF* locus is activated in tissues under oncogenic stresses, such as DNA damage and telomere shortening.  p16^Ink4a^ then acts to inhibit the kinase activities of cyclin D1 - CDK4, cyclin D2 - CDK4, and cyclin D3 - CDK6.  D-type cyclin and CDK complexes phosphorylate Rb/p105, when in the hypophosphorylated form Rb/p105 binds to E2F-1, 2, 3, and 4 blocking their activity as transcriptional activators.  E2F target genes are required for progression of the cell cycle and their transcriptional repression results in G1 cell cycle arrest and eventual replicative senescence.  p19^Arf^ interacts with p53 initiating p53-dependent cell death, or apoptosis.  p19^Arf^ can also slow the cell cycle and lead to senescence.  Similar to p16^Ink4a^, p19^Arf^ is not expressed in fetal stem cells but is found to increase in aging stem cells [76,79].

**Figure 2.**
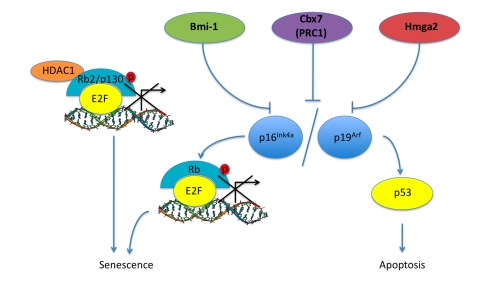
Pathways associated with aging in ASCs. Bmi-1, Cbx7 (PRC1), Hmga2 are proteins that have been shown to increase in expression levels in aging ASCs along with corresponding inhibition of the *INK4a/ARF* locus leading to a progression into senescence and apoptosis.  Rb2/p130 also shows an increase in senescent MSCs, this could be a result of HDAC1 - Rb2/p130 complex repressing E2F target gene transcription and initiating the senescence pathway.

To maintain their replicative and self-renewing potential stem cells have in place mechanisms to repress activation of cell death pathways.  Bmi-1 has been shown to promote self-renewal in stem cells by repressing the expression of p16^Ink4a^ and p19^Arf^ through negative regulation of the *INK4a/ARF* locus (Figure 2).  In *Bmi-1*^-/-^ neural stem cells, Molofsky and collegues found overexpression of p16^Ink4a^ and reduced rates of proliferation [81,82].  Park and collegues determined that Bmi-1 is essential for the self-renewal of HSCs.  They utilized a *Bmi-1*^-/-^ mouse model and showed that there was an increase in both p16^Ink4a^ and p19^Arf^ expression in HSCs leading to proliferation arrest and p53-dependent cell death.  The subsequent loss of p16^Ink4a^ expression in *Bmi-1*^-/-^cells was able to partially rescue the self-renewal capacity of the stem cells [82,83].

Bmi-1 is a Polycomb group (PcG) RING finger protein found to associate with the multiprotein PcG complex PRC1. PRC1 is a complex that maintains the repressive state of heterochromatin by modifying histone protein complexes.  It includes at least one paralogue of the Pcgf, Ring1, Phc and Cbx components as has been described [84].  PRC2 is the second complex that can associate with heterochromatin.  It is hypothesized that both PRC1 and PRC2 act in maintaining the heterochromatic structure necessary for stem cell self-renewal and with age it is believed that these mechanisms become inadequate and can lead to neoplastic transformation of stem cell populations.  In ESCs mutant for PRC2, there is a loss in the ability to maintain an un-differentiated state of self-renewal [85,86].  Futhermore, both PRC1 and PRC2 have been shown to be inter-dependent in their effects on stem cell self-renewal and cancer development [87,88].

In similar studies, other PcG proteins have been found to affect the *INK4a/ARF* locus in aging stem cells.  Jacobs and collegues show that Mel-18 and Cbx7 were found to regulate the *INK4a/ARF* locus [81].  Cbx7 is a PcG protein that helps form PRC1, this protein was independently found to extend the lifespan of primary human cell lines (Figure 2) [89].  When introduced into mouse fibroblasts, Cbx7 can result in immortalization of the cell line through the downregulation of the *INK4a/ARF* locus and interference with the p16^Ink4a^/Rb and p19^Arf^/p53 tumor suppressor pathways [89].

The age-dependent decline in ASC self-renewing capacity has been associated with various transcriptional regulators other than the PcG proteins discussed above.

Nishino and colleagues have recently discovered the involvement of Hmga2 in stem cell self-renewal [90].  Hmga2 is a transcriptional regulator and was found to be highly expressed in young neural stem cell populations in mice.  Its expression declines with age and is believed to be regulated by the microRNA let-7b. Induced expression of let-7b in stem cells with high levels of Hmga2, showed a decrease in Hmga2 levels in concordance with a decreased ability for self-renewal.  This corresponded to increased expression of p16^Ink4a^ and p19^Arf^ (Figure 2).  Furthermore, in mice deficient for *Hmga2* there were reduced stem cell numbers and self-renewal throughout the central and peripheral nervous system of fetal and young-adult mice [90].

Owing to the fact that the regulation of the ASC cell cycle is still not completely understood and because of the importance that the *INK4a/ARF* locus seems to play in aging ASCs, we performed a study on the senescence of rat MSCs *in vitro* looking specifically at the expression levels of Rb family proteins [91,92].  We observed that the induction of senescence was associated with a decrease in expression of genes involved in stem cell self-renewal, DNA-damage repair genes, p107 and Rb/p105.  However, Rb2/p130 expres-sion surprisingly increased during senescence in MSCs [92].  This suggested that Rb2/p130 plays a prominent role in either MSC specific aging and/or senescence.  It has previously been shown that Rb2/p130 can bind to HDAC1 repressing E2F-dependent gene expression, such as cyclin A, ultimately resulting in G_0_ growth arrest, supporting the possibility that Rb2/p130 has a more global role in cellular senescence (Figure 2) [92,93].

Over the past decade evidence has mounted in favor of the hypothesis that stem cell self-renewal is regulated through heterochromatin conformation under the control of PcG protein complexes.  This regulation appears to repress the *INK4a/ARF* locus, thus inhibiting the progression of tumor suppressive mechanisms such as senescence and apoptosis.  However, other regulatory mechanisms are present as seen with Hmga2, let-7b, and Rb2/p130, therefore the story is far from complete. The necessity for stem cells to maintain self-renewability appears to be in balance with the risk to enter into un-controlled proliferation and possibly cancer.  Further studies are necessary to clarify how these mechanisms play a role in the self-renewal, main- tenance, and senescence of stem cells.

### Concluding remarks

The area of stem cell research is vastly and rapidly expanding with the hope of its potential in therapeutic applications.  In this review we have discussed the current characterizations and understandings of ESCs and ASCs.  ESCs have been utilized not only to understand development, but to obtain a manipulative system that could be applied towards regenerative and disease based therapies. The first stem cell trial of this kind has just recently been approved by the Food and Drug Administration for Phase I clinical trial and is based upon the pre-clinical studies published in 2005 on hES cell-derived oligodendrocytes and their ability to remyelinate and restore function of the spinal chord in mice after injury [94]. This is the first example of a therapeutic potential that we have yet to reap and that will surely be expanded upon in the years to come.

Utilizing and understanding ESC differentiation *in vitro* may help elucidate the ASC populations, which until now have been tedious to isolate and lack accurate and universal cell markers.  Here we have outlined the current understanding of ESC populations and their regulation of the cell cycle.  More importantly we highlight the significance of maintaining a self-renewable population of ASCs and the regulation mechanisms that have been associated with this maintenance. The pathway that appears to be involved across the board is the *INK4a/ARF* CDK-inhibitor pathway, which regulates the two major mechanisms of cell death, senescence and apoptosis.  Both p16^Ink4a^ and p19^Arf^ have been found to be un-detectable in young stem cell populations but increase in aging populations, lending to the importance of these pathways in stem cell maintenance.  As we have discussed, there are multiple genetic and epigenetic factors that appear to be associated with the *INK4a/ARF* pathway regulation in ASCs, speaking to the complexity and the profundity of what we have yet to ascertain.

There is a cogent belief that organismal aging is linked to the aging and the loss of functional ASC populations.  The data discussed here support the role of senescence and apoptosis as self-regulative mechanisms in aging ASCs. Clarification and in-depth comprehension of these pathways may unveil needed therapeutic potentialities that could be applicative to both aging and age-related diseases.

## References

[1] Hayflick L (1965). The limited in vitro lifetime of human diploid cell strains. Exp Cell Res.

[2] Shamblott MJ, Axelman J, Wang S, Bugg EM, Littlefield JW, Donovan PJ, Blumenthal PD, Huggins GR, Gearhart JD (1998). Derivation of pluripotent stem cells from cultured human primordial germ cells. Proc Natl Acad Sci U S A.

[3] Thomson JA, Itskovitz-Eldor J, Shapiro SS, Waknitz MA, Swiergiel JJ, Marshall VS, Jones JM (1998). Embryonic stem cell lines derived from human blastocysts. Science.

[4] Martin GR, Evans MJ (1974). The morphology and growth of a pluripotent teratocarcinoma cell line and its derivatives in tissue culture. Cell.

[5] Martin GR, Evans MJ (1975). Differentiation of clonal lines of teratocarcinoma cells: Formation of embryoid bodies in vitro. Proc Natl Acad Sci U S A.

[6] Greider CW, Blackburn EH (1987). The telomere terminal transferase of tetrahymena is a ribonucleoprotein enzyme with two kinds of primer specificity. Cell.

[7] Hayflick L, Moorhead PS (1961). The serial cultivation of human diploid cell strains. Exp Cell Res.

[8] Harley CB, Futcher AB, Greider CW (1990). Telomeres shorten during aging of human fibroblasts. Nature.

[9] Allsopp RC, Vaziri H, Patterson C, Goldstein S, Younglai EV, Futcher B, Greider CW, Harley CB (1992). Telomere length predicts replicative capacity of human fibroblasts. Proc Natl Acad Sci U S A.

[10] Schopperle WM, DeWolf WC (2007). The TRA-1-60 and TRA-1-81 human pluripotent stem cell markers are expressed on podocalyxin in embryonal carcinoma. Stem Cells.

[11] Niwa H, Burdon T, Chambers I, Smith A (1998). Self-renewal of pluripotent embryonic stem cells is mediated via activation of STAT3. Genes Dev.

[12] Nichols J, Zevnik B, Anastassiadis K, Niwa H, Klewe-Nebenius D, Chambers I, Schöler H, Smith A (1998). Formation of pluripotent stem cells in the mammalian embryo depends on the POU transcription factor Oct4. Cell.

[13] Fuchs E, Segre JA (2000). Stem cells: A new lease on life. Cell.

[14] Brüstle O, Jones KN, Learish RD, Karram K, Choudhary K, Wiestler OD, Duncan ID, McKay RDG (1999). Embryonic stem cell-derived glial precursors: A source of myelinating transplants. Science.

[15] Keller G, Kennedy M, Papayannopoulou T, Wiles MV (1993). Hematopoietic commitment during embryonic stem cell differentiation in culture. Mol Cell Biol.

[16] Green H (1991). Cultured cells for the treatment of disease. Sci Am.

[17] Haas K, Johannes C, Geisen C, Schmidt T, Karsunky H, Blass-Kampmann S, Obe G, Möröy T (1997). Malignant transformation by cyclin E and Ha-Ras correlates with lower sensitivity towards induction of cell death but requires functional Myc and CDK4. Oncogene.

[18] Mumberg D, Haas K, Möröy T, Niedenthal R, Hegemann JH, Funk M, Müller R (1996). Uncoupling of DNA replication and cell cycle progression by human cyclin E. Oncogene.

[19] Rathjen J, Lake JA, Bettess MD, Washington JM, Chapman G, Rathjen PD (1999). Formation of a primitive ectoderm like cell population, EPL cells, from ES cells in response to biologically derived factors. J Cell Sci.

[20] Solter D, Skreb N, Damjanov I (1971). Cell cycle analysis in the mouse EGG-cylinder. Exp Cell Res.

[21] Hogan BLM, Beddington RSB, Constantini F, Lacy E (1994). Manipulating the Mouse Embryo. 2nd edition.

[22] Power MA, Tam PP (1993). Onset of gastrulation, morphogenesis and somitogenesis in mouse embryos displaying compensatory growth. Anat Embryol (Berl).

[23] Savatier P, Huang S, Szekely L, Wiman KG, Smaraut J (1994). Contrasting patterns of retinoblastoma protein expression in mouse embryonic stem cells and embryonic fibroblasts. Oncogene.

[24] Stead E, White J, Faast R, Conn S, Goldstone S, Rathjen J, Dhin-gra U, Rathjen P, Walker D, Dalton S (2002). Pluripotent cell division cycles are driven by ectopic Cdk2, cyclin A/E and E2F activities. Oncogene.

[25] Giacinti C, Giordano A (2006). RB and cell cycle progression. Oncogene.

[26] Kasten MM, Giordano A (1998). pRB and the Cdks in apoptosis and the cell cycle. Cell Death Differ.

[27] Fraichard A, Chassande O, Bilbaut G, Dehay C, Savatier P, Samarut J (1995). In vitro differentiation of embryonic stem cells into glial cells and functional neurons. J Cell Sci.

[28] Fluckinger AC, Marcy G, Marchand M, Négre D, Cosset FL, Mitalipov S, Wolf D, Savatier P, Dehay C (2006). Cell cycle features of primate embryonic stem cells. Stem Cells.

[29] Becker KA, Ghule PN, Therrien JA, Lian JB, Stein JL, van Wijnen AJ, Stein GS (2006). Self-renewal of human embryonic stem cells is supported by a shortened G1 cell cycle phase. J Cell Physiol.

[30] Nowell PC, Hirsch BE, Fox DH, Wilson DB (1970). Evidence for the existence of multipotential lympho-hematopoietic stem cells. J Cell Physiol.

[31] Thomas ED, Lochte HL, LU WC, Ferrebee JW (1957). Intravenous infusion of bone marrow in patients receiving radiation and chemotherapy. N Engl J Med.

[32] de Haan G (2002). Hematopoietic stem cells: self-renewing or aging. Cells Tissues Organs.

[33] Watt FM, Hogan BL (2000). Out of Eden: stem cells and their niches. Science.

[34] Potten CS (1998). Stem cells in gastrointestinal epithelium: numbers, characteristics and death. Philos Trans R Soc Lond B Biol Sci.

[35] Czyz J, Wiese C, Rolletschek A, Blyszczuk P, Cross M, Wobus AM (2003). Potential of embryonic and adult stem cells in vitro. Biol Chem.

[36] Tropepe V, Coles BL, Chiasson BJ, Horsford DJ, Elia AJ, McInnes RR, van der Kooy D (2000). Retinal stem cells in the adult mammalian eye. Science.

[37] Ramiya VK, Maraist M, Arfors KE, Schatz DA, Peck AB, Cornelius JG (2000). Reversal of insulin-dependent diabetes using islets generated in vitro from pancreatic stem cells. Nat Med.

[38] Theise ND, Saxena R, Portmann BC, Thung SN, Yee H, Chiriboga L, Kumar A, Crawford JM (1999). The canals of Hering and hepatic stem cells in humans. Hepatology.

[39] Okano H, Yochizaki T, Shimazaki T, Sawamoto K (2002). Isolation and transplantation of dopaminergic neurons and neural stem cells. Parkinsonism Relat Disord.

[40] Seale P, Asakura A, Rudnicki MA (2001). The potential of muscle stem cells. Dev Cell.

[41] Down JD, Boudewijn A, van Os R, Thames HD, Ploemacher RE (1995). Variations in radiation sensitivity and repair among different hematopoietic stem cell subsets following franctionated irradiation. Blood.

[42] Roobrouck VD, Ulloa-Montoya F, Verfaillie CM (2008). Self-renewal and differentiation capacity of young and aged stem cells. Exp Cell Res.

[43] Prockop DJ (1997). Marrow stromal cells as stem cells for nonhematopoietic tissues. Science.

[44] Pittenger MF, Mackay AM, Beck SC, Jaiswal RK, Douglas R, Mosca JD, Moorman MA, Simonetti DW, Craig S, Marshak DR (1999). Multilineage potential of adult human mesenchymal stem cells. Science.

[45] Jiang Y, Jahagirdar BN, Reinhardt RL, Schwartz RE, Keene CD, Ortiz-Gonzalez XR, Reyes M, Lenvik T, Lund T, Blackstad M, Du J, Aldrich S, Lisberg A (2002). Pluripotency of mesenchymal stem cells derived from adult marrow. Nature.

[46] Yoon YS, Wecker A, Heyd L, Park JS, Tkebuchava T, Kusano K, Hanley A, Scadova H, Qin G, Cha DH, Johnson KL, Aikawa R, Asahara T (2005). Clonally expanded novel multipotent stem cells from human bone marrow regenerate myocardium after myocardial infarction. J Clin Invest.

[47] Kogler G, Sensken S, Airey JA, Trapp T, Muschen M, Feldhahn N, Liedtke S, Sorg RV, Fischer J, Rosenbaum C, Greschat S, Knipper A, Bender J (2004). A new human somatic stem cell from placental cord blood with intrinsic pluripotent differentiation potential. J Exp Med.

[48] Kues WA, Peterson B, Mysegades W, Carnwath JW, Niemann H (2005). Isolation of murine and procine fetal stem cells from somatic tissue. Biol Reprod.

[49] De Coppi P, Bartsch G Jr (2007). , Siddiqui MM, Xu T, Santos CC, Perin L, Mostoslavsky G, Serre AC, Snyder EY, Yoo JJ, Furth ME, Soker S, Atala A. Isolation of amniotic stem cell lines with potential for therapy. Nat Biotechnol.

[50] D'Ippolito G, Diabira S, Howard GA, Menei P, Roos BA, Schiller PC (2004). Marrow-isolated adult multilineage inducible (MIAMI) cells, a unique population of postnatal young and old human cells with extensive expansion and differentiation potential. J Cell Sci.

[51] Dan YY, Riehle KJ, Lazaro C, Teoh N, Haque J, Campbell JS, Fausto N (2006). Isolation of multipotent progenitor cells from human fetal liver capable of differentiating into liver and mesenchymal lineages. Proc Natl Acad Sci U S A.

[52] Beltrami AP, Cesselli D, Bergamin N, Marcon P, Rigo S, Puppato E, D'Aurizio R, Verardo R, Piazza S, Pignatelli A, Poz A, Baccarani U, Damiani D (2007). Multipotent cells can be generated in vitro from several adult human organs (heart, liver, and bone marrow). Blood.

[53] Spradling A, Drummond-Barbosa D, Kai T (2001). Stem cells find their niche. Nature.

[54] Fuchs E, Tumbar T, Guasch G (2004). Socializing with the neighbors: stem cells and their niche. Cell.

[55] Reya T, Duncan AW, Ailles L, Domen J, Scherer DC, Willer K, Hintz L, Nusse R, Weissman IL (2003). A role for Wnt signaling in self-renewal of haematopoietic stem cells. Nature.

[56] Willert K, Brown JD, Danenberg E, Duncan AW, Weissman IL, Reya T, Yates JR III, Nusse R (2003). Wnt proteins are lipid-modified and can act as stem cell growth factors. Nature.

[57] Vescovi AL, Reynolds BA, Fraser DD, Weiss S (1993). bFGF regulates the proliferative fate of unipotent (neuronal) and bipotent (neuronal/astoglial) EGF-generated CNS progenitor cells. Neuron.

[58] Gritti A, Parati EA, Cova L, Frolichsthal P, Galli R, Wanke E, Faravelli L, Morassutti DJ, Roisen F, Nickel DD, Vescovi AL (1996). Multipotential stem cells from the adult mouse brain proliferate and self-renew in response to basic fibroblast growth factor. J Neurosci.

[59] Friedenshtein AIa (1982). Stromal bone marrow cells and the hematopoietic microenvironment. Arkh Patol.

[60] Caplan AI (1991). Mesenchymal stem cells. J Orthop Res.

[61] Ivanova NB, Dimos JT, Schaniel C, Hackney JA, Moore KA, Lemischka IR (2003). A stem cell molecular signature. Science.

[62] Anisimov SV, Terasov KV, Tweedie D, Stern MD, Wobus AM, Boheler KR (2002). SAGE identification of gene transcripts with profiles unique to pluripotent mouse R1 embryonic stem cells. Genomics.

[63] Ramalho-Santos M, Yoon S, Matsuzaki Y, Mulligan RC, Melton DA (2002). ‘Stemness': transcriptional profiling of embryonic and adult stem cells. Science.

[64] Rossi DJ, Bryder D, Zahn JM, Ahlenius H, Sonu R, Wagers AJ, Weissman IL (2005). Cell intrinsic alterations underlie haematopoietic stem cell aging. Proc Natl Acad Sci U S A.

[65] Rossi DJ, Bryder D, Seita J, Nussenzweig A, Hoeijmakers J, Weissman IL (2007). Deficiencies in DNA damage repair limits the function of haematopoietic stem cells with age. Nature.

[66] Chambers SM, Shaw CA, Gatza C, Fisk CJ, Donehower LA, Goodell MA (2007). Aging haematopoietic stem cells decline in function and exhibit epigenetic dysregulation. Plos Biol.

[67] Sherr CJ, Roberts JM (1995). Inhibitors of mammalian G1 cyclin-dependent kinases. Genes Dev.

[68] Campisi J, d'Adda di Fagagna F (2007). Cellular senescence: when bad things happen to good cells. Nat Rev Mol Cell Biol.

[69] Beauséjour CM, Krtolica A, Galimi F, Narita M, Lowe SW, Yaswen P, Campisi J (2003). Reversal of human cellular senescence: roles of the p53 and p16 pathways. EMBO J.

[70] Guo K, Wang J, Andrés V, Smith RC, Walsh K (1995). MyoD-induced expression of p21 inhibits cyclin-dependent kinase activity upon myocyte terminal differentation. Mol Cell Biol.

[71] Halevy O, Novitch BG, Spicer DB, Skapek SX, Rhee J, Hannon GJ, Beach D, Lassar AB (1995). Correlation of terminal cell cycle arrest of skeletal muscle with induction of p21 by MyoD. Science.

[72] Cheng T, Rodrigues N, Shen H, Yang Y, Dombkowski D, Sykes M, Scadden DT (2000). Hematopoietic stem cell quiescence maintained by p21cip1/waf1. Science.

[73] Kwon YH, Jovanovic A, Serfas MS, Kiyokawa H, Tyner AL (2002). P21 functions to maintain quiescence of p27-deficient hepatocytes. J Biol Chem.

[74] Sang L, Coller HA, Roberts JM (2008). Transcriptional repressor HES1. Science.

[75] Molofsky AV, Slutsky SG, Joseph NM, He S, Pardal R, Krishnamurthy J, Sharpless NE, Morrison SJ (2006). Increasing p16Ink4a expression decreased forebrain progenitors and neurogenesis during ageing. Nature.

[76] Maslov AY, Barone TA, Plunkett RJ, Pruitt SC (2004). Neural stem cell detection, characterization, and age-related changes in the subventricular zone of mice. J Neurosci.

[77] Lowe SW, Sherr CJ (2003). Tumor suppression by Ink4a-Arf: progress and puzzles. Curr Opin Genet Dev.

[78] Krishnamurthy J, Torrice C, Ramsey MR, Kovalev GI, Al-Regaiey K, Su L, Sharpless NE (2004). Ink4a/Arf expression is a biomarker of aging. J Clin Invest.

[79] Zindy F, Quelle DE, Roussel MF, Sherr CJ (1997). Expression of the p16Ink4a tumor suppressor versus other INK4 family members during mouse development and aging. Oncogene.

[80] Serrano M (2000). The INK4a/ARF locus in murine tumorigenesis. Carcinogenesis.

[81] Jacobs JJL, Kieboom K, Marino S, DePinho RA, von Lohuizen M (1999). The oncogene and polycomb-group gene bmi-1 regulates cell proliferation and senescence through the ink4a locus. Nature.

[82] Molofsky AV, Pardal R, Iwashita T, Park I, Clarke MF, Morrison SJ (2003). Bmi-1 dependence distinguishes neural stem cell self-renewal from progenitor proliferation. Nature.

[83] Park I, Qian D, Kiel M, Becker MW, Pihalja M, Weissman IL, Morrison SJ, Clarke MF (2003). Bmi-1 is required for maintenance of adult self-renewing haematopoietic stem cells. Nature.

[84] Sauvageau M, Sauvageau G (2008). Polycomb group genes: keeping stem cell activity in balance. PLOS Biol.

[85] Lee TI, Jenner RG, Boyer LA, Guenther MG, Levine SS, Kumar RM, Chevalier B, Johnstone SE, Cole MF, Isono K, Koseki H, Fuchikami T, Abe K (2006). Control of developmental regulators of Polycomb in human embryonic stem cells. Cell.

[86] Boyer LA, Plath K, Zeitlinger J, Brambrink T, Medeiros LA, Lee TI, Levine SS, Wernig M, Tajornar A, Ray MK, Bell GW, Otte AP, Vidal M (2006). Polycomb complexes repress developmental regulators in murine embryonic stem cells. Nature.

[87] Sparmann A, van Lohuizen M (2006). Polycomb silencers control cell fate, development and cancer. Nat Rev Cancer.

[88] Stock JK, Giadrossi S, Casanova M, Brookes E, Vidal M, Koseki H, Brockdorff N, Fisher AG, Pombo A (2007). Ring1-mediated ub-iquitination of H2A restrains poised RNA polymerase II at bivalent genes in mouse ES cells. Nat Cell Biol.

[89] Gil J, Bernard D, Martínez D, Beach D (2004). Polycomb CBX7 has a unifying role in cellular lifespan. Nat Cell Biol.

[90] Nishino J, Kim I, Chada K, Morrison SJ (2008). Hmga2 promotes neural stem cell self-renewal in young but not old mice by reducing p16Ink4a and p19Arf expression. Cell.

[91] Galderisi U, Cipollaro M, Giordano A (2006). The retinoblastoma gene is involved in multiple aspects of stem cell biology. Oncogene.

[92] Galderisi U, Helmold H, Squillaro T, Alessio N, Komm N, Khadang B, Cipollaro M, Bohn W, Giordano A (2008). In vitro senescence of rat mesenchymal stem cells is accompanied by downregulation of stemness-related and DNA damage repair genes. Stem Cells Dev.

[93] Siegler P, De Luca A, Bagella L, Giordano A (1998). The COOH-terminal region of pRb2/p130 binds to histone deacetylase 1 (HDAC1), enhancing transcription repression of the E2F-dependent cyclin A promoter. Cancer Res.

[94] Keirstead HS, Nistor G, Bernal G, Totoiu M, Cloutier F, Sharp K, Seward O (2005). Human embryonic stem cell-derived oligodendrocyte progenitor cell transplants remyelinate and restore locomotion after spinal chord injury. J Neurosci.

